# Scrotal abscess with a rare cause

**DOI:** 10.4103/0971-9261.57707

**Published:** 2009

**Authors:** Khizer Mansoor, Ram Samujh, Yasen Fayez AlAlayet

**Affiliations:** Department of Pediatric Surgery, Riyadh Medical Complex, Riyadh, KSA; 1 Department of Pediatric Surgery, Advanced Pediatric Center, Post Graduate Institute of Medical Education and Research, Chandigarh, India

**Keywords:** Acute scrotum, scrotal abscess, scrotal swelling

## Abstract

A 4-year-old boy presented with a short history of right-sided acute scrotal pain and swelling. At exploration, pus was found in the hemiscrotum but no local cause could be found. Further exploration showed the pus coming through a patent processus vaginalis from a collection in the right iliac fossa secondary to acute appendicitis.

## INTRODUCTION

Common causes of acute scrotum include acute testicular torsion or one of its appendages, acute epididymoorchitis, incarcerated inguinoscrotal hernia or idiopathic scrotal edema or rarely scrotal abscess.[1] Appendicitis presenting as scrotal abscess is rare.

## CASE REPORT

A 4-year-old boy presented with a 3-day history of right-sided scrotal pain and swelling, which had gradually increased. The pain as well as swelling had started gradually. There was no history of trauma. Patient had vomited once on day 2 and also developed fever a day before admission. There was no history of sudden onset of pain, trauma, mumps or urinary complaints. He had been treated by antipyretics only.

On examination, he was distressed because of pain and had a body temperature of 38.2°C. Local examination showed a red, hot, tender and erythematous right hemiscrotum. The cremasteric reflex was absent. Left side was normal. Abdominal examination revealed mild tenderness in the right lower quadrant only. The results of remaining systemic examination were within normal limits.

Investigations revealed a white cell (WBC) count of 12,800, with 85% neutrophils with left shift. The findings of remaining laboratory investigations were within normal limits. Scrotal ultrasonography revealed thickened epididymis and fluid collection with internal echoes. Blood flow to the testis was normal and contralateral side was also normal.

With the diagnosis of scrotal abscess, an exploration was undertaken under general anesthesia. About 1.5-2 cm^3^ of thick pus was removed and only mild edema of the epididymis was noted. After cleaning the pus, no primary source of pus could be detected locally and it appeared to be coming from the neck of the scrotum. A fine, size-8 tube was introduced in search of a source and it passed smoothly up into the abdomen and pus started to come freely from this (on retrospective questioning the mother clearly denied the presence of a hernia). With the idea of an intra-abdominal source, an exploratory laparotomy was undertaken through a right-sided Lanz incision. A severely inflamed retrocecal appendix was found with localized peritonitis. Appendectomy was done and abdomen closed with a drain in place. Scrotum was closed with loose interrupted sutures without attempting to ligate the patent processus vaginalis (PPV) because of severe local edema. Patient showed an uneventful and quick recovery.

The patient is doing well at 1-year follow-up with normal scrotal ultrasound and no inguinal swelling.

## DISCUSSION

Scrotal abscess is a rare condition in the pediatric age group. It can present in any age group and a few cases have been reported even in the neonatal age group.[2] The most common cause is postneglected testicular torsion or necrotizing epididymoorchitis. Other causes include infection of the hydrocele or tuberculous infection. A very rare cause is scrotal collection as presentation of acute appendicitis, with less than 25 cases reported in literature. Most patients who develop acute scrotal signs due to appendicular pathology have a PPV.[1] Less than one-third of these patients have a clinical hernia before presentation.

The presentation is usually of severe scrotal pain, redness, heat, tenderness and systemic toxicity including fever and leukocytosis. Patient may or may not have vomiting. At times, lower abdominal pain is present but this is attributed to the scrotal pathology rather than as a cause of the scrotal abscess. Clinical examination is not very helpful because of the tenderness. Ultrasound examination usually reveals mild fluid with internal echoes or a hypoechoic lesion with normal or swollen scrotal contents.[3] As the most important differential is torsion of either the testis or one of its appendages, a surgical exploration is almost always needed.

Usually upon exploration, turbid fluid is encountered.[4] If the fluid recollects after mopping and no local pathology is detected, this should raise the suspicion of an intra-abdominal cause. Sometimes the tip or the entire appendix may be seen in the sac.[5] Usually, a PPV is found. An abdominal exploration may show an acute appendicitis,[6] a perforated appendix[7] or a retroperitoneal abscess.[8] Although mostly this disease occurs on the right side, left-sided acute scrotum has also been reported due to appendicular pathology.[9] Sometimes the diagnosis is missed and recognized later because of the continuing toxicity. Abdominal sonography, computerized tomography and magnetic resonance imaging all have been used to diagnose the intra-abdominal pathology. Drainage of pus, appendectomy and closure of the PPV during the same sitting offers satisfactory results.[5]

Acute appendicitis presenting as scrotal abscess is a very rare entity. It should always be considered in the differential diagnosis of an acute scrotum. If turbid fluid or frank pus is found during exploration of an acute scrotum without a local cause, attempts should be made to find a PPV. Local toilet, ligation of the PPV and dealing with the abdominal cause offers satisfactory results in this rare condition.
